# Preadult polytoxicomania—strong environmental underpinnings and first genetic hints

**DOI:** 10.1038/s41380-021-01069-2

**Published:** 2021-04-07

**Authors:** Agnes A. Steixner-Kumar, Vinicius Daguano Gastaldi, Jan Seidel, Albert Rosenberger, Martin Begemann, Hannelore Ehrenreich

**Affiliations:** 1 Clinical Neuroscience, Max Planck Institute of Experimental Medicine, Göttingen, Germany; 2 Department of Genetic Epidemiology, University Medical Center, Georg-August-University, Göttingen, Germany; 3 Department of Psychiatry and Psychotherapy, University Medical Center, Georg-August-University, Göttingen, Germany

**Keywords:** Addiction, Schizophrenia, Genetics

## Abstract

Considering the immense societal and personal costs and suffering associated with multiple drug use or “polytoxicomania”, better understanding of environmental and genetic causes is crucial. While previous studies focused on single risk factors and selected drugs, effects of early-accumulated environmental risks on polytoxicomania were never addressed. Similarly, evidence of genetic susceptibility to particular drugs is abundant, while genetic predisposition to polytoxicomania is unexplored. We exploited the GRAS data collection, comprising information on N~2000 deep-phenotyped schizophrenia patients, to investigate effects of early-life environmental risk accumulation on polytoxicomania and additionally provide first genetic insight. Preadult accumulation of environmental risks (physical or sexual abuse, urbanicity, migration, cannabis, alcohol) was strongly associated with *lifetime* polytoxicomania (*p*  = 1.5 × 10^−45^; OR = 31.4), *preadult* polytoxicomania with OR = 226.6 (*p* = 1.0 × 10^−33^) and *adult* polytoxicomania with OR = 17.5 (*p* = 3.4 × 10^−24^). Parallel accessibility of genetic data from GRAS patients and N~2100 controls for genome-wide association (GWAS) and phenotype-based genetic association studies (PGAS) permitted the creation of a novel multiple GWAS–PGAS approach. This approach yielded 41 intuitively interesting SNPs, potentially conferring liability to preadult polytoxicomania, which await replication upon availability of suitable deep-phenotyped cohorts anywhere world-wide. Concisely, juvenile environmental risk accumulation, including cannabis and alcohol as starter/gateway drugs, strongly predicts polytoxicomania during adolescence and adulthood. This pivotal message should launch more effective sociopolitical measures to prevent this deleterious psychiatric condition.

## Introduction

Substance use disorders and multiple drug consumption are frequent in the general population and recurrent comorbidities of schizophrenia and other neuropsychiatric disorders [1–3]. Multiple drug use, also called polytoxicomania (the preferred term throughout this manuscript), is defined as the use of ≥3 drugs in a given period and associated with a range of negative behavioral, physical and mental health outcomes. These comprise higher psychological distress, impaired neuropsychological function, increased anxiety and depression, unsafe sexual behavior, greater risk for infectious diseases, and enhanced mortality, including suicides [4, 5]. Considering the large societal and personal costs and suffering associated with polytoxicomania, its high prevalence in teenagers (8–18%) [6–8] is alarming. Yet, its underlying causes are largely unknown. Previous studies have proposed that different individual (e.g., gender, school grades, ethnicity, depression, personality traits), familial (e.g., parental monitoring, inner-family conflicts), and other social factors (e.g., connectedness, drug use by peers) can increase the risk for polytoxicomania [9–12], but systematic investigations of a broader spectrum of risk factors, especially in large samples, are rare.

The preadult accumulation of environmental risk factors is a potent predictor of various health outcomes and negative behaviors, including earlier age at schizophrenia onset [13], reduced global functioning and higher severity of psychopathology [14, 15], boosted violent-aggressive behavior [16], unfavorable child development [17], enhanced use of tobacco, alcohol and other drugs as well as delinquency and suicidal behavior in students [18]. The predisposing risks for polytoxicomania have mostly been studied in isolation, and reports on how cumulative risk affects polytoxicomania are scarce (e.g., [19, 20]). Therefore, the current study investigates for the first time effects of preadult environmental risk accumulation on polytoxicomania based on the unique high-quality deep-phenotyping data of GRAS (Göttingen Research Association for Schizophrenia) [21].

Moreover, we aimed at shedding first light on possible genetic risk factors predisposing to polytoxicomania by exploiting the extensive genotyping information of GRAS. Past studies focused on genetic associations with single drugs (e.g., [22–24]). Few reports addressed any genetic underpinnings of polytoxicomania, concentrating on single genes, or performing genome-wide association studies (GWAS) on lifetime drug experimentation [25–27]. Even though twin studies suggest genetic liability to substance use disorders in adolescence [28], no study has explored preadult polytoxicomania on a genome-wide level likely due to world-wide missing databases on this condition which impedes any kind of replication study. Therefore, our novel multiple GWAS–PGAS (phenotype-based genetic association studies) approach utilizes extensive phenotyping information to improve the confidence with which genetic signals from single nucleotide polymorphisms (SNPs) in relatively small (*n* < 3500), but deep-phenotyped samples, can be identified.

We show here that environmental risk accumulation, including physical abuse, sexual abuse, migration, urbanicity, and the use of gateway drugs, cannabis, and alcohol [29, 30], strongly promotes polytoxicomania. Further, we support existing evidence that preadult polytoxicomania induces aggressive and criminal behavior later in life. Finally, we deliver first hints for a genetic susceptibility to preadult polytoxicomania, which ultimately require independent replication upon availability of suitable cohorts anywhere worldwide.

## Subjects and methods

### Subjects

The extended GRAS data collection consists of N~2000 schizophrenic and schizoaffective subjects (according to DSM-IV-TR). Ethics Committees of Georg-August-University, Göttingen, and participating centers across Germany approved GRAS, complying with Helsinki Declaration. All patients (and/or legal representatives) gave written informed consent. For genetic association analyses, also healthy blood donors (*N* = 2111; age 33.68 ± 12.21 years, 58.46% males) were included. Complete phenotypical information was available for *N* = 1904 schizophrenia/schizoaffective patients (40.28 ± 13.11 years, 66.02% males), complete genotype information for *N* = 1718 individuals (40.44 ± 13.07 years, 66.94% males) after exclusion of individuals due to relatedness, ancestral outlier status, and missing phenotype data. We note that individuals were only included in phenotypic or genetic analyses if unambiguous information on environmental risk, drug use/autistic traits/suicidal behavior was obtainable, explaining some *N* number variation.

### Phenotyping

#### Environmental risk

Environmental risk factors, including physical abuse, sexual abuse, migration, urbanicity, cannabis use and problematic alcohol use before age of 18 years and onset of schizophrenia, were operationalized as described in detail earlier [13, 16]. Additional environmental risk factors (for evaluation of autistic traits only) included perinatal complications, season of birth, early neurotrauma, paternal age, number of infections (positive serology) [13, 16].

#### Polytoxicomania

Medical reports and SCID-I allowed assessment of current and previous drug use. Drug classes included cocaine, opioids, hallucinogens, amphetamines, ecstasy, barbiturates, benzodiazepines, and inhalants. In the environmental risk study, use of ≥3 drugs was coded as polytoxicomania, use of ≤2 drugs as non-polytoxicomanic behavior. For the multiple GWAS–PGAS approach, exact definitions of polytoxicomania for each GWAS are provided in display items. Note that caffeine was not considered as “drug”.

#### Other outcomes

*Premorbid intelligence* was measured by Mehrfachwahl–Wortschatz–Intelligenztest-B (MWT-B [31]), corrected for age and medication (CPZ; chlorpromazine equivalents) using standardized residuals from linear regression. *Suicidality*: Suicide attempts served as readout. *Autistic traits*: PANSS Autism Severity Score (PAUSS) [32] was used to assess severity.

### Genotyping

Genotyping was performed using a semi-custom Axiom® myDesign^TM^ genotyping array (Affymetrix, Santa Clara, CA, USA), based on a CEU (Caucasian residents of European ancestry from Utah, USA) marker backbone including 518,722 SNPs, and a custom marker set including 102,537 SNPs (described in detail [33]). A total of 530,316 autosomal variants passed quality control, had minor allele frequency (MAF) > 0.05, were in Hardy–Weinberg equilibrium (HWE; *p* > 0.001) and therefore included in genetic analyses.

### Genetic association analysis

From each pair of related individuals (second to third-degree relatives, PIHAT > 0.185) one individual was randomly excluded. In healthy blood donor-patient pairs, priority was given to patient samples. Ancestral outliers were excluded based on principal component analysis (PCA; principal component1 (PC1 ± 3 SD) and PC2 ± 5 SD). PCs were calculated on LD-pruned marker set (long-range LD-regions removed) of 96,836 autosomal SNPs. In all association tests (SNP test: logistic regression; gene-based test: PC regression), sex and first 10 PCs were entered as covariates to control for population stratification.

### Human expression data

Human tissue expression data (standardized) was downloaded from Harmonizome [34] (https://maayanlab.cloud/Harmonizome/dataset/GTEx+Tissue+Gene+Expression+Profiles, accessed 23/11/2020).

### Statistical analysis

Calculations of relatedness, principal components, genetic association analyses, and LD-based clumping (index variant *p* value threshold=0.01) of GWAS results were performed in PLINK v1.90 [35], gene-based association tests in MAGMA v1.07b [36]. Chi-square tests and Fisher’s exact tests were applied to test for categorical associations. Cochran–Armitage or Jonckheere–Terpstra tests (permutations *n* = 20,000) were employed for trends. Levene’s test was used to assess homogeneity of variances. In case of homoscedasticity, Welch’s, Games–Howell test, or Welch’s ANOVA (R-package “onewaytests”) were applied for group comparisons and post-hoc tests with continuous outcomes (Bonferroni-corrected). In all instances where mathematical assumptions could have been violated, robust statistical tests were carried out to exclude a negative impact of such violations on statistical outcomes. Calculation of variance inflation factors (VIF) assured absence of multicollinearity between predictor variables (all VIF < 1.2), except when smoking was included (thus rejecting it as predictor). Odds ratio (OR) was calculated using R-package “DescTools” or exact logistic regression with permutation (PROC LOGISTIC) when cell count was equal to 0 (SAS 9.4). Except for OR calculations, all statistical tests and workflows were performed in R v3.5.2 [37] or IBM SPSS Statistics for Windows, Version 25.

## Results

### Preadult polytoxicomania and aggressive behavior

**Fig. 1:**
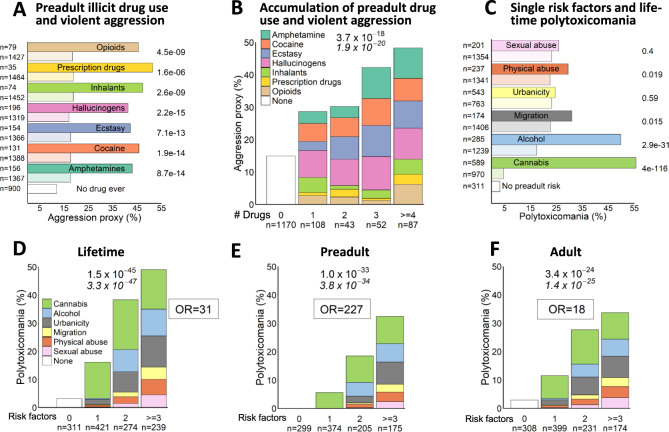
Environmental influence on behavioral phenotypes. **A** Associations of preadult illicit drug use with violent aggressive behavior as measured by an aggression proxy (developed in Mitjans et al. [16]). Columns in dark colors denote preadult users of the respective drug; light colors indicate non-users of this specific drug. Note that drug use shown is not exclusive, i.e., a person that consumed opioids might also have used inhalants and thus appear in both columns as user; a person that used cocaine but not ecstasy will appear as non-user for ecstasy. Group “No drug ever” refers to lifetime non-consumption of any illicit drug. Left side: *N* numbers. Right side: *P* values (Chi²-test, two-sided) comparing users and non-users. Note that in this panel, cannabis, alcohol, nicotine and caffeine are not considered. **B** Step-wise increase in aggression proxy with the number of drug classes used in early life. Colors in bars represent respective drug classes. **C** Associations of single preadult environmental risk factors and lifetime polytoxicomania. Columns in dark colors denote individuals exposed to respective risk factor, light colors refer to individuals not exposed to this specific risk (not necessarily devoid of any risk at all). Note that risks shown are not exclusive, i.e., many individuals carry more than 1 risk factor. **D**–**F** Accumulation of preadult environmental risk factors leads to stepwise increase in lifetime polytoxicomania (**D**), exclusive preadult polytoxicomania (**E**) and polytoxicomania appearing exclusively later in life (**F**). Colors indicate respective risk factors (**B**), (**D**–**F**): *n* numbers below bars, (**A**), (**B**) on left side. Chi²-test *p* values (two-sided) on top of graph (**B**), (**D**–**F**) or right side (**A**), (**B**); Cochran–Armitage test *p* values (two-sided) (**B**, **C**), (**E**) underneath in italics. OR: Odds Ratio.

We first explored the predictive potential of individual illicit drugs for aggressive behavior using a violent aggression proxy (Fig. 1A) [16]. The proportion of violently aggressive individuals increased step-wise with the number of illicit drugs consumed before age 18 years (Fig. 1B): Nearly 50% of individuals consuming ≥4 drugs in early life, i.e., who were clearly polytoxicomanic at a young age, showed violent aggression as compared to just 15% in the group of individuals, who did not use any illicit drug before age 18 (extreme group comparison OR = 5.3, 95% CI [3.4–8.3]).

### Contrasting drug use in non-polytoxicomanic and polytoxicomanic individuals

A comparison of lifetime patterns of illicit drug use amongst non-polytoxicomanic (<3 drugs) and polytoxicomanic (≥3 drugs) individuals showed a highly diverse drug use in the latter group (Supplementary Table 1). While the vast majority of non-polytoxicomanic individuals never had contact with illicit/hard drugs in their life (>95% for any drug), the majority of polytoxicomanic individuals had used most of the respective drug classes at least once, e.g., only 13% and 24% did not have any contact with cocaine or ecstasy, respectively. Furthermore, frequency of use in polytoxicomanic individuals varied substantially between drug classes, e.g., although only 18% never tried hallucinogens, a minority of individuals (~3%) report using it (almost) daily. This combination makes it difficult to entirely exclude certain drug preferences at the individual level. Overall, however, polytoxicomanic individuals in our sample displayed a highly diverse and frequent use of drugs, without systematic preference. This clearly discriminates them from individuals classified here as non-polytoxicomanic.

### Accumulated preadult environmental risk predicts polytoxicomania

Next, we evaluated the prognostic value of individual preadult environmental risks for polytoxicomania. Whereas all factors by themselves showed some tendency, our “secondary” environmental risks, alcohol, and cannabis, revealed already here their expected role as gateway/starter drugs (Fig. 1C). The following accumulation analysis showed that the higher the number of preadult risk factors, the higher the likelihood of polytoxicomania over lifetime (extreme group comparison OR = 31.4, 95% CI [15.9–61.9]) (Fig. 1D). We next stratified by preadult polytoxicomania (≥3 drugs consumed before age 18 years/onset of schizophrenia, Fig. 1E) and adult polytoxicomania (≥3 drugs consumed throughout life, but after age 18 years/onset of schizophrenia, Fig. 1F). This resulted in extreme probabilities for developing polytoxicomania preadult with OR = 226.6 (extreme group comparison; 95% CI [50.8-∞]) and adult with OR = 17.5 (extreme group comparison; 95% CI [8.4–36.4]), strongly pointing to a causal relationship between preadult environmental risk and polytoxicomania.

### Preadult environmental risk accumulation predicts polytoxicomania also without inclusion of starter drugs in the model

To alleviate concerns of potential circularity when including the starter drugs cannabis and alcohol as secondary environmental risks of polytoxicomania, we omitted them on a trial basis, using only 4 risk factors as predictors (urbanicity, migration, physical abuse, sexual abuse) (Supplementary Fig 1A–C). Reassuringly, the overall pattern of risk accumulation predicting polytoxicomania remained identical even though the effect was expectedly less pronounced.

### Lack of evidence of an appreciable role of tobacco as gateway drug

Smoking prevalence in our sample was very high, with 85% of individuals reporting to have smoked at least once in their life. Of those, 80% started before age 18 and onset of schizophrenia. Thus, although nicotine may be often consumed before other drugs, due to its high prevalence (and thus “non-specificity”) its predictive value for use of illicit or hard drugs is rather limited. For example, whereas 37% and 34% of our individuals, who consumed cannabis (mostly also smokers) or had problematic alcohol use (either in isolation or in combination with other risk factors) in preadulthood, respectively, developed preadult polytoxicomania, “only” 19% of young smokers (including those with a combination of tobacco with cannabis and possibly other risk factors) progressed to preadult polytoxicomania.

Of note, multicollinearity excluded smoking as valid predictor. In fact, there was a very strong relationship between tobacco and cannabis use (cannabis is mostly smoked with tobacco). In our sample only 2.5% of individuals, who used cannabis at young age, never smoked before age 18 and onset of schizophrenia. They consumed cannabis in cookies or similar. Vice versa, 53% of all preadult smokers in our sample also report usage of cannabis. Nonetheless, when including smoking in a purely exploratory fashion as additional risk factor, the accumulation model expectedly does not improve (Supplementary Fig 1D–F). Interestingly, when smoking was the only risk factor an individual was exposed to, it did not increase the probability of being polytoxicomanic, questioning a substantial role of tobacco by itself as starter drug.

### Risk load comparison in individuals with or without polytoxicomania and role of cannabis and alcohol as starter drugs re-visited

**Fig. 2:**
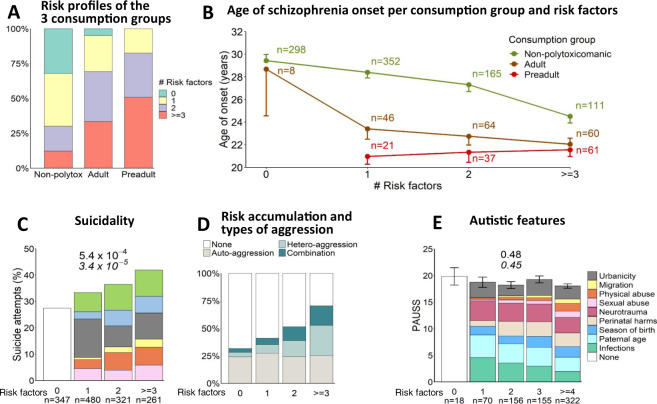
Environmental risk load and behavioral consequences. **A** Risk factor distribution in the 3 different consumption groups: non-polytoxicomanic individuals (lifetime), exclusively adult polytoxicomanic, and exclusively preadult polytoxicomanic individuals, shows a clearly elevated risk load in (preadult) polytoxicomanic individuals. **B** Relation of number of preadult environmental risk factors and drug consumption behavior with age at schizophrenia onset. Mean±SEM. **C** Stair pattern increase in percentage of individuals who attempted to commit suicide with number of preadult environmental risk factors; legend of risk factors as in (Fig. 1D). **D** Distribution of auto- and heteroaggressive behavior shows that autoaggression (suicide attempts) increases with the number of risk factors only in individuals who show heteroaggressive behavior as well. **E** Not all behavioral traits increase with environmental risk: No association of preadult environmental risk factors and autistic features in adulthood as quantified by PAUSS (developed in Kästner et al. [32]). Colors indicate respective risk factors. Mean±SEM (**C**), (**E**): *n* numbers below bars. Chi²-test (**C**) or one-way ANOVA (**E**) *p* values (two-sided) on top of graph; two-sided Cochran–Armitage (**C**) and Jonckheere–Terpstra trend tests (**E**) underneath in italics.

Risk load in preadult polytoxicomanic subjects was immense (Fig. 2A). Nearly 50% of all individuals showing preadult polytoxicomania had experienced ≥3 risk factors before the age of 18 years/onset of schizophrenia. In the group of adult polytoxicomania, 34% had ≥3 risk factors in contrast to only 12% of individuals without polytoxicomania. All preadult polytoxicomanic individuals (100%) experienced at least 1 risk factor in early life. Likewise, in the adult polytoxicomanic group, only 5% of individuals had no risk factor before adulthood. In contrast, 32% of individuals in the non-polytoxicomanic group were “risk-free” (Fig. 2A).

Subsequently, we investigated in detail the distribution of individual risk factors in the consumption groups. There was a strong group difference regarding preadult cannabis exposure (*p* = 4.59e–93, Table 1), with 100% of preadult polytoxicomanic individuals (irrespective of risk numbers) having consumed cannabis at early age. Among adult polytoxicomanic individuals, this number dropped to 91.7%, 87.5%, and 69.6% in the risk groups with ≥3, 2, or 1 risk factors, respectively. In comparison, within the non-polytoxicomanic group, the percentage of individuals with cannabis use in early life was lower (68.1%, 47.3%, 14.7%, respectively), yet still substantial. The prevalence of preadult problematic alcohol use followed a similar but less pronounced pattern. In addition, migration and physical abuse differed between consumption groups (Table 1). Overall, the most frequent risk factor was urbanicity (43.1%), followed by cannabis (38.3%) and physical abuse (16.5%) (Table 1).

**Table 1:** Prevalence of individual risk factors stratified by consumption group and number of risks.

		*n* (%)					
Group	# Risk factors	Cannabis	Alcohol	Urbanicity	Migration	Physical abuse	Sexual abuse
Preadult	**1**	21 (100.0)	0 (0.0)	0 (0.0)	0 (0.0)	0 (0.0)	0 (0.0)
Adult		32 (69.6)	2 (4.3)	7 (15.2)	1 (2.2)	3 (6.5)	1 (2.2)
Non-polytox		52 (14.7)	31 (8.8)	191 (54.1)	12 (3.4)	30 (8.5)	37 (10.5)
Preadult	**2**	38 (100.0)	20 (52.6)	10 (26.3)	2 (5.3)	5 (13.2)	1 (2.6)
Adult		56 (87.5)	21 (32.8)	29 (45.3)	7 (10.9)	9 (14.1)	6 (9.4)
Non-polytox		79 (47.3)	38 (22.8)	105 (62.9)	28 (16.8)	47 (28.1)	37 (22.2)
Preadult	**≥3**	61 (100.0)	42 (70.0)	43 (81.1)	20 (32.8)	22 (36.1)	16 (26.7)
Adult		55 (91.7)	35 (59.3)	41 (73.2)	19 (31.7)	24 (40.0)	22 (36.7)
Non-polytox		77 (68.1)	57 (50.4)	98 (88.3)	44 (38.6)	63 (55.3)	44 (38.9)
Total^a^		**471 (38.3)**	**246 (20.0)**	**524 (43.1)**	**133 (10.8)**	**203 (16.5)**	**164 (13.3)**
*p (χ²)*		***4.59e−93 (425.23)***	***1.72e−26 (118.65)***	*0.58 (1.08)*	***0.001 (13.61)***	***0.042 (6.35)***	*0.421 (1.73)*

Bold values indicate significant differences (*p* < 0.05) in the prevalence of respective risk factors between the three consumption groups (across number of risk factors).

^a^Calculation of total percentage also included individuals with 0 risk factors. Underlined *p* values remain significant after Bonferroni correction (*p* < 0.05/6 = 0.008). Two-sided Chi²-test applied.

Individuals in the consumption groups differed from each other with respect to age (*p* = 6.26e−62), age at disease onset (*p* = 5.87e−38), and premorbid intelligence (corrected for age and medication, *p* = 0.002), but not medication (*p* = 0.38; Table 2). Premorbid intelligence of preadult polytoxicomanic individuals was significantly lower than that of adult polytoxicomanic (*p* = 0.01) and non-polytoxicomanic (*p* = 0.02), but did not differ between the latter two groups (*p* > 0.99). A two-way ANOVA confirmed the main effects of environmental risk group and consumption group on age of onset without interaction between the two groups (environmental risks: *p* = 8.71e−08, consumption: *p* = 4.66e−12, risks×consumption: *p* = 0.23). Figure 2B depicts the substantial impact of polytoxicomanic behavior on age at disease onset. In the non-polytoxicomanic individuals, the age at disease onset step-wise decreases with the number of risk factors (*p* = 1.73e−08). In preadult and adult polytoxicomanic individuals, an increasing number of risk factors had no appreciable further influence on the already early age at disease onset (*p* = 0.89 and *p* = 0.30, respectively).

**Table 2:** Sociodemographic and disease-related outcomes stratified by consumption group.

Total	Non-polytox	Adult	Preadult	*p*
Mean (SD)				
**Age (years)**				
39.59 (12.71)	41.98 (12.73)	34.00 (9.81)	28.86 (6.86)	**6.26e−62**
*n* = 1559	*n* = 1192	*n* = 213	*n* = 154	
**Age at disease onset (years)**				
25.95 (8.81)	27.21 (9.22)	22.60 (6.17)	20.82 (4.66)	**5.87e−38**
*n* = 1531	*n* = 1170	*n* = 210	*n* = 151	
**Chlorpromazine equivalents**				
679.25 (670.09)	669.81 (678.96)	740.58 (694.05)	669.71 (558.51)	0.38
*n* = 1513	*n* = 1161	*n* = 202	*n* = 150	
**Premorbid intelligence MWT-B-corrected**				
0.04 (1.00)	0.05 (1.04)	0.13 (0.96)	−0.16 (0.70)	**0.002**
*n* = 1440	*n* = 1101	*n* = 194	*n* = 145	

*MWT-B* Mehrfachwahl–Wortschatz–Intelligenztest-B; Premobid intelligence: Standardized residuals corrected for Chlorpromazine equivalents and age. Sample sizes vary because of missing data. Bold values indicate significant differences between means of the three consumption groups. Underlined *p* values remain significant after Bonferroni correction (*p* < 0.05/4 = 0.0125). Two-sided Welch’s ANOVA (age, age at disease onset, premorbid intelligence) or ANOVA (Chlorpromazine equivalents) applied.

### Effects of accumulated preadult environmental risk on other behavioral outcomes

In view of these striking results, we explored the possibility that preadult environmental risk accumulation might be a potent predictor of any other behavioral outcome as well. Building on our previous findings of violent-aggressive behavior increasing with preadult environmental risk [16], we investigated its impact on autoaggressive behavior in form of suicidality. Indeed, with rising number of environmental risks, we also observed a surge in percentage of individuals with suicidal thoughts (*p* = 0.012, Cochran–Armitage test; OR = 1.5, 95% CI [1.1–2.2], extreme group comparison), suicidal plans (*p* = 0.004, Cochran–Armitage test; OR = 1.7, 95% CI [1.2–2.3], extreme group comparison) and suicide attempts (Fig. 2C; *p* = 3.3e−05, Cochran–Armitage test; OR = 2.0, 95% CI [1.4–2.8], extreme group comparison). This surge, however, appears to be explained only by individuals with additional heteroaggression (Fig. 2D).

As another behavioral readout, we assessed whether preadult risk accumulation can also predict severity of autistic features in adulthood (Fig. 2E). For this approach, we even included more risk factors, previously reported to play a role in autistic behavior, i.e., perinatal complications, early neurotrauma, season of birth, paternal age, and number of postnatal infections. Interestingly, we did not observe any association of autistic traits with any environmental risk factor, neither individually (all *p* > 0.05) nor in accumulation.

### Genetic susceptibility to preadult polytoxicomania

**Fig. 3:**
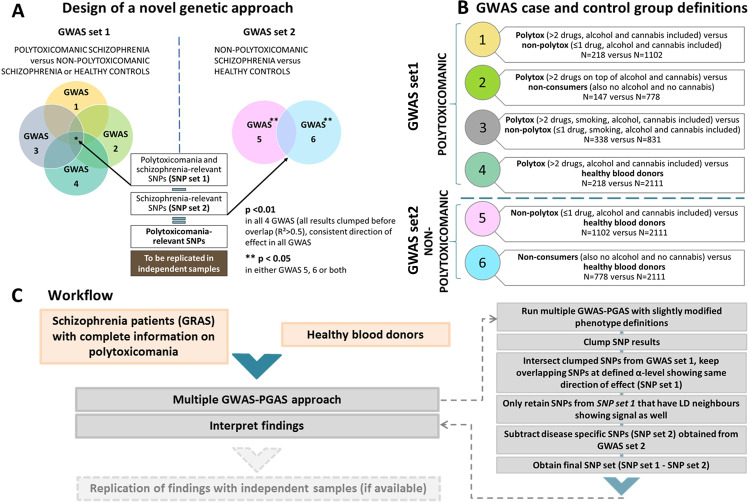
Novel multiple GWAS–PGAS approach. **A** Overview of the analysis design. GWAS set 1 to obtain SNP set 1: 4 GWAS contrasting polytoxicomanic versus non-polytoxicomanic individuals (including healthy individuals) with slightly varied phenotype definitions (details in (**B**)) to identify SNPs that show consistent associations (*p* < 0.01) in all 4 GWAS. These SNPs are considered polytoxicomania and/or schizophrenia-associated. GWAS set 2 to obtain SNP set 2: SNPs associated exclusively with schizophrenia, but not polytoxicomania, resulting from GWAS 5 and/or 6 (*p* < 0.05) are subtracted from SNP set 1 (polytoxicomania and/or schizophrenia-associated), yielding the final set of polytoxicomania-relevant SNPs. **B** Diagram showing exact phenotype definitions and sample sizes per group for GWAS 1–6. **C** Detailed workflow of the novel GWAS–PGAS approach including clumping procedure to reduce number of SNPs in the final set.

**Fig. 4:**
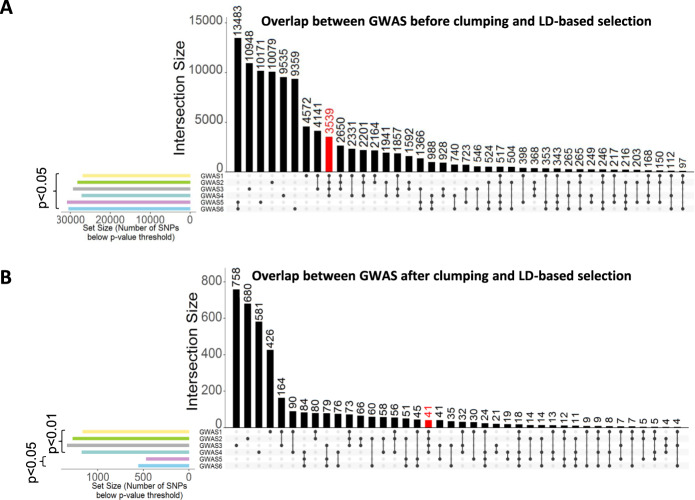
Intersection of SNP results before and after clumping. **A** Intersection raw results before clumping of associated SNPs from all 6 GWAS. Black bars represent the number of intersecting SNPs below *p* value threshold 0.05. Dots indicate the respective GWAS for which the number of intersecting SNPs was calculated. Columns with single dots indicate SNPs unique to the corresponding GWAS. GWAS 1-4 and GWAS 5-6 show strong overlap within each other. Importantly, as indicated by the red bar, the largest intersection size when overlapping 4 GWAS is between GWAS 1-4 (polytoxicomania and/or schizophrenia GWAS). These SNPs show no association in GWAS 5-6. The novel approach applied to raw GWAS results would thus yield 3539 SNPs. Colored bars on the left indicate the number of associated SNPs per individual GWAS, i.e., the set size. **B** Intersections of associated SNPs from all 6 GWAS building on clumped SNP results and considering LD linkage neighbor signals. Black bars represent the number of intersecting SNPs below the given *p* value threshold on the left. Dots indicate the respective GWAS for which the number of intersecting SNPs was calculated. Columns with single dots indicate SNPs unique to the corresponding GWAS. Again, the intersection of SNPs associated in GWAS 1-4, but not GWAS 5-6, is larger than for any other combinations of 4 GWAS and yields now a strongly reduced set of 41 polytoxicomania-associated SNPs (red bar). Colored bars on the left indicate the number of associated SNPs per individual GWAS, i.e., the set size.

In order to overcome the challenges associated with detecting genetic associations in relatively small, but deeply phenotyped, high-quality samples, we developed the novel multiple GWAS–PGAS approach to discover potential first associations of common genetic variants (SNPs) with preadult polytoxicomania. As depicted in Fig. 3A, B, this approach rests on multiple GWAS for polytoxicomania with minor variations in phenotype definitions. Supplementary Fig. 2 presents Manhattan plots for GWAS 1-6. The underlying working hypotheses of the workflow described in Fig. 3C, presume that (i) true genetic signals should be stable across (minor to moderate) changes in sample composition, (ii) true genetic signals are more likely to have neighbors in linkage disequilibrium which show a signal, too (but not entirely excluding that false do as well), and (iii) the here identified genetic associations with polytoxicomania should be independent of schizophrenia diagnosis (although pleiotropic risk genes exist [38] but are a separate matter). Figure 4A, B depicts the overlap structure between different GWAS results at given *p* value threshold. Importantly, GWAS 1-4 (set1) have the highest number of shared SNPs among each other in the raw (*n* = 3539-red; Fig. 4A) as well as the clumped/LD-linked results (*n* = 41-red; Fig. 4B)—convincingly visualized by Jaccard index matrix (Supplementary Fig 3). Notably, clumping and usage of linkage information considerably condense the number of final polytoxicomania-associated SNPs, identifying 41 potentially relevant SNPs (at *p* < 0.01, with LD neighbors *p* < 0.05; graphically displayed as red dots in Manhattan plots, Supplementary Fig 2). Out of these, 28 were located in gene-coding regions (±10 kb, Supplementary Table 2). Interestingly, 15 of these genes (out of 20 with human tissue expression data available on Harmonizome [34]) are strongly expressed in brain and/or kidney (Supplementary Fig 4). In addition, gene-wide association results (MAGMAv1.07b) yielded 11 genes potentially associated with preadult polytoxicomanic behavior (Supplementary Table 3). We abstained from deeper interpretation of these genes at this stage because of the still required replication. Known associations, however, listed in Supplementary Tables 2 and 3, are promising.

## Discussion

This study discovered a prominent association of polytoxicomania with early accumulation of environmental risk factors, comprising urbanicity, migration, sexual abuse, physical abuse and, as secondary risks, alcohol, and cannabis. In particular, preadult polytoxicomania is >220 times more likely to develop in subjects exposed to ≥3 risks. In addition, polytoxicomania in later life occurs with higher probability after adolescent accumulation of negative environmental impact. Thus, our data show that multiple drug use has obviously remarkable roots in preadult risk factor buildup. In perfect agreement with previous reports (e.g., [39–41]), we find clear escalation in percentage of violent behavior with increasing numbers of drugs consumed. Strikingly, the number of accumulated risk factors appears to be more informative than the type of environmental risk factor. Nonetheless, our data strongly underline the well-known significance of cannabis and alcohol as gateway/starter drugs [29, 42–46].

In fact, all individuals with preadult polytoxicomania reported using cannabis. This confirms a tremendous association between cannabis and preadult polytoxicomania, even though not ultimately proving causality. A more causal perspective is perhaps offered by our data on polytoxicomania arising in adulthood. In adult polytoxicomanic individuals, the preadult risk factors temporally clearly precede the occurrence of polytoxicomania. Also in this group, cannabis is the by far most frequent preadult risk factor (occurring in 70–92% of individuals). Considering this extraordinary impact of cannabis, legalization and commercialization of its use will certainly increase not only incidence and prevalence of psychosis [47–53], but also of polytoxicomania.

Importantly, smoking as the sole risk factor did not increase the chance of developing polytoxicomania and did not influence our accumulation model further. Along these lines, heavy increase in tobacco consumption is highly frequently observed in alcoholics upon alcohol abstinence. This phenomenon is called *‘*“*Suchtverlagerung*” or shift in addictive behaviors and is not associated with generation of polytoxicomania (but causes other severe problems [54]), again emphasizing the “non-specificity” of nicotine consumption in this regard. Nevertheless, it cannot be entirely excluded that tobacco has a minor role as “co-gateway” drug together with cannabis (as cannabis use is tightly associated with smoking status).

Cannabis and alcohol are sometimes seen as potential consequences of early-life stress, like physical and/or sexual abuse, considered as some sort of self-treatment [55]. However, the VIF for all variables in our model was low (all VIF < 1.2), indicating that there was no considerable multicollinearity between the risk factor variables. This suggests an independent input of each variable to the model including independent contributions of cannabis/alcohol and stress-related variables such as physical or sexual abuse.

We show that suicidality, as a severe form of autoaggression, follows a similar stair-pattern, but only in individuals exhibiting heteroaggression as well. Based on all these results, one could speculate that any kind of behavioral traits might be shaped by preadult environmental risk exposure; nevertheless, we did not see any respective influence of the here determined risks on the severity of autistic phenotypes in adulthood. It seems that autistic features are predominantly genetically induced [56, 57]. However, impact of intrauterine damage, e.g., viral infections, on development of autism is well established [58] and, like other potential postnatal risk factors, not recorded here.

The present findings are derived from a large, deeply phenotyped sample of schizophrenic subjects with uniquely comprehensive information on multiple drug consumption, but their translation to the general population has still to ensue with some caution. Nevertheless, preadult polytoxicomania in our sample occurs way before onset of the disease, and may thus reflect general mechanisms of damage to the juvenile brain that predispose to multiple drug consumption rather than any direct association with mental illness. The view of essential generalizability receives support by our studies on violent aggression, where we had the chance to include two general population samples that revealed the same stair-pattern of aggression development with risk accumulation as the 4 independent schizophrenia cohorts [16]. The earlier onset of mental illness in polytoxicomanic individuals shown here emphasizes another devastating role of multiple drug use on brain functions and on outcome in mental disease. Mechanistically, this phenomenon as well as the observed behavioral alterations are likely mediated by epigenetic changes that occur relatively non-specifically in response to accumulated risk factor exposure in early life. These epigenetic changes apparently increase, as others and we have reported previously, the propensity for later substance abuse, depression, violent aggression, and a range of other negative health outcomes [16, 59–61].

On top of the remarkable environmental impact on polytoxicomania, we provide first evidence of a genetic predisposition to this behavioral abnormality as extracted from our novel GWAS–PGAS approach. This novel approach was only possible based on the comprehensive and precise information on drug consumption in our sample, which allowed the accurate classification of just mildly varying phenotypes for respective GWAS. We defined strict criteria for this GWAS–PGAS approach, which is potentially applicable to other genetic association studies as well. Our procedure is grounded on the working hypotheses that true genetic signals remain stable despite mild modifications in phenotype definition and are likely not isolated but in linkage disequilibrium with related signals, and that genetic associations with the target phenotype (here: polytoxicomania) are independent of any other disease diagnosis (here: schizophrenia). Diagnoses-overlapping, i.e., pleiotropic genes [38] were purposely excluded here. The mild variation of phenotype definitions allowed us to identify genetic associations that are more specific for polytoxicomania and not “contaminated” by associations stemming solely from single substance abuse.

We ultimately extracted an interesting set of markers as potential “bouquet of common genetic denominators” for juvenile polytoxicomania, many of them strongly expressed in brain and kidney. Unfortunately, no comparable sample of deeply phenotyped polytoxicomanic individuals is presently available for replication studies—despite intensive worldwide search—but would be crucial to have. Nevertheless, the nature of the obtained first genetic results seems encouraging.

## Supplementary information

Supplementary Tables 1-3

Supplementary Figures 1-4

## Data Availability

The data supporting the findings of this study are not publicly available because of strict obligation to human data protection laws. The contained information could lead to identification of subjects and compromise research participants’ privacy and consent. Additional information and summary statistics are available on request from the corresponding author (HE).
