# Supplementary material for: Preadult polytoxicomania—strong environmental underpinnings and first genetic hints
Source: Mol Psychiatry. 2021 Apr 7;26(7):3211–22. doi: 10.1038/s41380-021-01069-2 (PMC8505259; doi:10.1038/s41380-021-01069-2)
Supplement: Supplementary file 1 — Supplementary Tables 1-3 [file 41380_2021_1069_MOESM1_ESM.docx]

**SUPPLEMENTARY TABLES**

**Preadult polytoxicomania – strong environmental underpinnings**

**and first genetic hints**

Agnes A. Steixner-Kumar^1^, Vinicius Daguano Gastaldi^1^, Jan Seidel^1^,

Albert Rosenberger², Martin Begemann^1,3^, and Hannelore Ehrenreich^1^*

^1^Clinical Neuroscience, Max Planck Institute of Experimental Medicine, Göttingen, Germany

² Department of Genetic Epidemiology, University Medical Center,

Georg-August-University, Göttingen, Germany

^3^Department of Psychiatry and Psychotherapy, University Medical Center,

Georg-August-University, Göttingen, Germany

**Running head:** Origins of polytoxicomania

**Key words:** Multiple drug use, schizophrenia, environmental risk, cannabis, alcohol, genome-wide association study (GWAS), phenotype-based genetic association study (PGAS), suicidality, autism

***Correspondence:**

**Prof. Hannelore Ehrenreich, MD, DVM**

Clinical Neuroscience

Max Planck Institute of Experimental Medicine

Hermann-Rein-Str.3

37075 Göttingen, GERMANY

Phone +49-551-3899628

Fax +49-551-3899670

E-Mail: **ehrenreich@em.mpg.de**

**Supplementary Table file contains:**

- Supplementary Tables1-3

***Please note*: In addition to this file, a Supplementary Figures file is available containing:**

- Supplementary Figures1-4

| \| Supplementary Table1. Lifetime prevalence (%) of illicit drug use amongst non-polytoxicomanic and polytoxicomanic individuals. \| \| \| \| \| \| \| \| \| --- \| --- \| --- \| --- \| --- \| --- \| --- \| --- \| \| Non-polytoxicomanic individuals (lifetime) \| \| \| \| \| \| \| \| \| Frequency of lifetime use \| (almost) daily \| several times  per week \| 1 per month to  1 per week \| 3-11 times  per year \| \| up to 2 times  per year \| never \| \| Drug \| **% Individuals using a particular drug at given frequency** \| \| \| \| \| \| \| \| Cocaine \| 0.08 \| 0.08 \| 0.17 \| 0.08 \| 3.67 \| \| 95.91 \| \| Opioids \| 0.5 \| 0.08 \| 0.08 \| 0 \| 1.5 \| \| 97.84 \| \| Hallucinogens \| 0 \| 0 \| 0.25 \| 0 \| 4.28 \| \| 95.48 \| \| Ecstasy \| 0 \| 0 \| 0.08 \| 0 \| 2.31 \| \| 97.6 \| \| Amphetamines \| 0.25 \| 0.17 \| 0.33 \| 0 \| 1.58 \| \| 97.67 \| \| Prescription drugs \| 1.48 \| 0.08 \| 0.25 \| 0.08 \| 0.16 \| \| 97.94 \| \| Inhalants \| 0.33 \| 0 \| 0.08 \| 0 \| 0.66 \| \| 98.92 \|   Polytoxicomanic individuals (lifetime) | | | | | | |
| --- | --- | --- | --- | --- | --- | --- | --- | --- | --- | --- | --- | --- | --- | --- | --- | --- | --- | --- | --- | --- | --- | --- | --- | --- | --- | --- | --- | --- | --- | --- | --- | --- | --- | --- | --- | --- | --- | --- | --- | --- | --- | --- | --- | --- | --- | --- | --- | --- | --- | --- | --- | --- | --- | --- | --- | --- | --- | --- | --- | --- | --- | --- | --- | --- | --- | --- | --- | --- | --- | --- | --- | --- | --- | --- | --- | --- | --- | --- | --- | --- | --- | --- | --- | --- | --- | --- | --- | --- | --- | --- | --- | --- | --- | --- |
| Frequency of lifetime use | (almost)  daily | several times  per week | 1 per month to  1 per week | 3-11 times  per year | up to 2 times  per year | never |
| Drug | **% Individuals using a particular drug at given frequency** | | | | | |
| Cocaine | 15.31 | 5.94 | 7.81 | 3.44 | 54.69 | 12.81 |
| Opioids | 17.87 | 1.25 | 2.82 | 0.31 | 32.29 | 45.45 |
| Hallucinogens | 2.79 | 2.23 | 6.7 | 3.63 | 66.76 | 17.88 |
| Ecstasy | 5.57 | 24.46 | 11.76 | 2.48 | 31.58 | 24.15 |
| Amphetamines | 22.67 | 14.67 | 11.67 | 2 | 33 | 16 |
| Prescription drugs | 11.78 | 0.82 | 1.1 | 1.37 | 9.86 | 75.07 |
| Inhalants | 1.19 | 0.59 | 0.59 | 0.59 | 24.63 | 72.4 |

| **Supplementary Table2. Top 41 polytoxicomania-associated SNPs** | | | | | | | | |
| --- | --- | --- | --- | --- | --- | --- | --- | --- |
| **SNP** | **Chr** | **Position** | **A1>A2** | **Gene**  **(±10kb)** | **Region** | **Annotation** | **Full name of gene** | **Previously reported GWAS associations (p<1x10^-5) with SNP or gene (psychiatric phenotypes only)** |
| [**rs41270726**](http://genome.ucsc.edu/cgi-bin/hgTracks?db=hg19&position=chr1:167633269-167633270) | 1 | 167633270 | G>T | [RCSD1](http://dec2013.archive.ensembl.org/Homo_sapiens/geneview?gene=ENSG00000198771) | protein coding | intronic | RCSD Domain Containing 1 | None |
| [**rs12038205**](http://genome.ucsc.edu/cgi-bin/hgTracks?db=hg19&position=chr1:83897415-83897416) | 1 | 83897416 | C>T | - | intergenic | - | - | None |
| [**rs1840328**](http://genome.ucsc.edu/cgi-bin/hgTracks?db=hg19&position=chr2:165785585-165785586) | 2 | 165785586 | A>G | [SLC38A11](http://dec2013.archive.ensembl.org/Homo_sapiens/geneview?gene=ENSG00000169507) | protein coding | intronic | Solute Carrier Family 38 Member 11 | None |
| [**rs16849964**](http://genome.ucsc.edu/cgi-bin/hgTracks?db=hg19&position=chr2:165802504-165802505) | 2 | 165802505 | C>T | [SLC38A11](http://dec2013.archive.ensembl.org/Homo_sapiens/geneview?gene=ENSG00000169507) | protein coding | intronic | Solute Carrier Family 38 Member 11 |  |
| [**rs1115381**](http://genome.ucsc.edu/cgi-bin/hgTracks?db=hg19&position=chr2:234505284-234505285)**■** | 2 | 234505285 | T>C | - | intergenic | - | - | None |
| [**rs7592624**](http://genome.ucsc.edu/cgi-bin/hgTracks?db=hg19&position=chr2:234602905-234602906) | 2 | 234602906 | G>A | [UGT1A complex region](http://dec2013.archive.ensembl.org/Homo_sapiens/geneview?gene=ENSG00000241635) | protein coding | - | UDP Glucuronosyltransferase Family 1 Member A Complex Locus | None |
| [**rs3821165**](http://genome.ucsc.edu/cgi-bin/hgTracks?db=hg19&position=chr2:36610068-36610069) | 2 | 36610069 | G>A | [CRIM1](http://dec2013.archive.ensembl.org/Homo_sapiens/geneview?gene=ENSG00000150938) | protein coding | intronic | Cysteine Rich Transmembrane BMP Regulator 1 | Irritability^1^ (gene) |
| [**rs10490237**](http://genome.ucsc.edu/cgi-bin/hgTracks?db=hg19&position=chr2:50377831-50377832) | 2 | 50377832 | A>C | [NRXN1](http://dec2013.archive.ensembl.org/Homo_sapiens/geneview?gene=ENSG00000179915) | protein coding | intronic | Neurexin 1 | Depressive affect subcluster and symptoms^1-3^ (SNP, gene*), neuroticism^1,2,4^(SNP, gene*), well-being^2^ (SNP, gene*), alcohol dependence^5^ (gene), initiation*/ever*/current/never/regular smoking^1,6-8^ (gene), nicotine dependence symptoms^9^ (gene), miserableness, fed-up and guilty feelings^1,10^* (gene), schizophrenia^11^ (gene), temperament/novelty seeking^12^ (gene) |
| [**rs10198285**](http://genome.ucsc.edu/cgi-bin/hgTracks?db=hg19&position=chr2:61979565-61979566) | 2 | 61979566 | A>G | - | intergenic | - | - | None |
| [**rs10496205**](http://genome.ucsc.edu/cgi-bin/hgTracks?db=hg19&position=chr2:77493706-77493707) | 2 | 77493707 | C>T | [LRRTM4](http://dec2013.archive.ensembl.org/Homo_sapiens/geneview?gene=ENSG00000176204) | protein coding | intronic | Leucine Rich Repeat Transmembrane Neuronal 4 | ADHD^13^ (gene), past/ever smoking, alcohol with meals* and ever cannabis*^2^ (gene), cannabis use^14^ (gene), number of sexual partners*^7^ (gene), risky behaviors 1^st^ PC*^7^ (gene), schizophrenia^11,15,16^ (gene), insomnia*^17^ (gene) |
| [**rs9862216**](http://genome.ucsc.edu/cgi-bin/hgTracks?db=hg19&position=chr3:147368415-147368416) | 3 | 147368416 | G>A | - | intergenic | - | - | None |
| [**rs16852499**](http://genome.ucsc.edu/cgi-bin/hgTracks?db=hg19&position=chr3:168326056-168326057) | 3 | 168326057 | G>A | [EGFEM1P](http://dec2013.archive.ensembl.org/Homo_sapiens/geneview?gene=ENSG00000206120) | pseudo-gene | intronic | EGF Like And EMI Domain Containing 1, Pseudogene | Epilepsy^18^ (gene), response to paliperidone in schizophrenia^19^ (gene) |
| [**rs11131889**](http://genome.ucsc.edu/cgi-bin/hgTracks?db=hg19&position=chr4:179851964-179851965)**■** | 4 | 179851965 | T>C | - | intergenic | - | - | None |
| [**rs10027326**](http://genome.ucsc.edu/cgi-bin/hgTracks?db=hg19&position=chr4:19056853-19056854) | 4 | 19056854 | A>G | - | intergenic | - | - | None |
| [**rs2703898**](http://genome.ucsc.edu/cgi-bin/hgTracks?db=hg19&position=chr4:38511325-38511326) | 4 | 38511326 | G>A | [RP11-83C7.2](http://dec2013.archive.ensembl.org/Homo_sapiens/geneview?gene=ENSG00000249534) | lincRNA | intronic | - | None |
| [**rs10517941**](http://genome.ucsc.edu/cgi-bin/hgTracks?db=hg19&position=chr4:66694544-66694545)**■** | 4 | 66694545 | C>A | - | intergenic | - | - | None |
| [**rs13126941**](http://genome.ucsc.edu/cgi-bin/hgTracks?db=hg19&position=chr4:7372805-7372806) | 4 | 7372806 | C>T | [SORCS2](http://dec2013.archive.ensembl.org/Homo_sapiens/geneview?gene=ENSG00000184985) | protein coding | intronic | Sortilin Related VPS10 Domain Containing Receptor 2 | Alcohol dependence* and withdrawal symptoms*^5,20^ (gene), insomnia^1,21^(gene), tense*^1^ (gene), attention function in ADHD^22^ (gene), depressive/manic episodes in bipolar disorder^23^ (gene), neuroticism^24^ (gene), anorexia nervosa^25^ (gene), response to antidepressants^26^ (gene), coffee consumption^27^ (gene) |
| [**rs17284960**](http://genome.ucsc.edu/cgi-bin/hgTracks?db=hg19&position=chr5:163623501-163623502) | 5 | 163623502 | C>T | [CTC-207P7.1](http://dec2013.archive.ensembl.org/Homo_sapiens/geneview?gene=ENSG00000253331) | lincRNA | intronic | - | None |
| [**rs6926569**](http://genome.ucsc.edu/cgi-bin/hgTracks?db=hg19&position=chr6:150375210-150375211) | 6 | 150375211 | A>G | [ULBP3](http://dec2013.archive.ensembl.org/Homo_sapiens/geneview?gene=ENSG00000131019) | protein coding | - | UL16 Binding Protein 3 | None |
| [**rs390661**](http://genome.ucsc.edu/cgi-bin/hgTracks?db=hg19&position=chr6:95090660-95090661) | 6 | 95090661 | C>T | - | intergenic | - | - | None |
| [**rs11764575**](http://genome.ucsc.edu/cgi-bin/hgTracks?db=hg19&position=chr7:115611308-115611309) | 7 | 115611309 | G>A | [TFEC](http://dec2013.archive.ensembl.org/Homo_sapiens/geneview?gene=ENSG00000105967) | protein coding | intronic | Transcription Factor EC | Chronotype/morningness*^17,28,29^ (gene) |
| [**rs2286248**](http://genome.ucsc.edu/cgi-bin/hgTracks?db=hg19&position=chr7:14216608-14216609) | 7 | 14216609 | G>A | [DGKB](http://dec2013.archive.ensembl.org/Homo_sapiens/geneview?gene=ENSG00000136267) | protein coding | intronic | Diacylglycerol Kinase Beta | Alcohol dependence^5^ (gene), Sensitivity*^1,10^ (gene), morningness^17^ (gene), major depression^30^ (gene), nicotine dependence symptoms^9^ (gene). |
| [**rs10279025**](http://genome.ucsc.edu/cgi-bin/hgTracks?db=hg19&position=chr7:21247252-21247253) | 7 | 21247253 | T>C | [RN7SL542P](http://dec2013.archive.ensembl.org/Homo_sapiens/geneview?gene=ENSG00000243633) | misc RNA | - | - | None |
| [**rs501344**](http://genome.ucsc.edu/cgi-bin/hgTracks?db=hg19&position=chr8:103114572-103114573) | 8 | 103114573 | G>A | [NCALD](http://dec2013.archive.ensembl.org/Homo_sapiens/geneview?gene=ENSG00000104490) | protein coding | intronic | Neurocalcin Delta | Sleep duration^1,17,31^ (gene), major depression and alcohol dependence comorbidity^32^ (gene), coffee consumption^33^ (gene) |
| [**rs10098626**](http://genome.ucsc.edu/cgi-bin/hgTracks?db=hg19&position=chr8:34888913-34888914)**■** | 8 | 34888914 | G>A | - | intergenic | - | - | None |
| [**rs2169385**](http://genome.ucsc.edu/cgi-bin/hgTracks?db=hg19&position=chr8:9206677-9206678) | 8 | 9206678 | G>A | [RP11-115J16.1](http://dec2013.archive.ensembl.org/Homo_sapiens/geneview?gene=ENSG00000254235) | lincRNA | intronic | - | Neuroticism^4^ (SNP) |
| [**rs4743569**](http://genome.ucsc.edu/cgi-bin/hgTracks?db=hg19&position=chr9:105414407-105414408) | 9 | 105414408 | A>G | [LINC00587](http://dec2013.archive.ensembl.org/Homo_sapiens/geneview?gene=ENSG00000204250) | lincRNA | intronic | - | None |
| [**rs2122582**](http://genome.ucsc.edu/cgi-bin/hgTracks?db=hg19&position=chr9:107009130-107009131) | 9 | 107009131 | T>C | - | intergenic | - | - | None |
| [**rs7851907**](http://genome.ucsc.edu/cgi-bin/hgTracks?db=hg19&position=chr9:9006518-9006519) | 9 | 9006519 | C>G | [PTPRD](http://dec2013.archive.ensembl.org/Homo_sapiens/geneview?gene=ENSG00000153707) | protein coding | intronic | Protein Tyrosin Phosphatase Receptor Type D | Response to amphetamine^34^ (gene), insomnia*^1,21^ (gene), alcohol dependence*^5^ (gene), baseline positive affect factor score*^34^ (gene), chronotype/morningness/sleep duration*^1,2,29^ (gene), depressive symptoms^2^ (gene), well-being^2^ (gene), ADHD and conduct disorder^35^ (gene), smoking initiation*^6^ (gene), opioid use cessation^36^ (gene), restless leg syndrome^37^ (gene), epilepsy remission after treatment^38^ (gene), migraine^39^ (gene) |
| [**rs11258725**](http://genome.ucsc.edu/cgi-bin/hgTracks?db=hg19&position=chr10:5720423-5720424) | 10 | 5720424 | T>A | [FAM208B](http://dec2013.archive.ensembl.org/Homo_sapiens/geneview?gene=ENSG00000108021) | protein coding | - | Transcription Activation Suppressor Family Member 3 | None |
| [**rs7101264**](http://genome.ucsc.edu/cgi-bin/hgTracks?db=hg19&position=chr10:91293183-91293184)**■** | 10 | 91293184 | C>A | [SLC16A12](http://dec2013.archive.ensembl.org/Homo_sapiens/geneview?gene=ENSG00000152779) | protein coding | intronic | Solute Carrier Family 38 Member 10 | None |
| [**rs35724134**](http://genome.ucsc.edu/cgi-bin/hgTracks?db=hg19&position=chr11:116306906-116306907) | 11 | 116306907 | C>A | - | intergenic | - | - | None |
| [**rs599905**](http://genome.ucsc.edu/cgi-bin/hgTracks?db=hg19&position=chr11:128544890-128544891) | 11 | 128544891 | A>G | [RP11-744N12.3](http://dec2013.archive.ensembl.org/Homo_sapiens/geneview?gene=ENSG00000245008) | antisense | intronic | - | None |
| [**rs10501240**](http://genome.ucsc.edu/cgi-bin/hgTracks?db=hg19&position=chr11:40695478-40695479)**■** | 11 | 40695479 | C>G | [LRRC4C](http://dec2013.archive.ensembl.org/Homo_sapiens/geneview?gene=ENSG00000148948) | protein coding | intronic | Leucine Rich Repeat Containing 4C | Risky behaviors 1^st^ PC*^7^ (gene), ever/regular smoker^6,7^(gene), age at smoking initiation*^7^ (gene), social support^1^ (gene) |
| [**rs12284778**](http://genome.ucsc.edu/cgi-bin/hgTracks?db=hg19&position=chr11:80829873-80829874) | 11 | 80829874 | T>C | - | intergenic | - | - | None |
| [**rs11111457**](http://genome.ucsc.edu/cgi-bin/hgTracks?db=hg19&position=chr12:103525944-103525945) | 12 | 103525945 | G>T | [RP11-328J6.1](http://dec2013.archive.ensembl.org/Homo_sapiens/geneview?gene=ENSG00000257703) | antisense | intronic | - | None |
| [**rs1863879**](http://genome.ucsc.edu/cgi-bin/hgTracks?db=hg19&position=chr12:105046249-105046250) | 12 | 105046250 | G>A | [CHST11](http://dec2013.archive.ensembl.org/Homo_sapiens/geneview?gene=ENSG00000171310) | protein coding | intronic | Carbohydrate Sulfotransferase 12 | Cannabis dependence^40^ (gene), response to major depression treatment^41^ (gene) |
| [**rs12316797**](http://genome.ucsc.edu/cgi-bin/hgTracks?db=hg19&position=chr12:43160743-43160744) | 12 | 43160744 | T>G | - | intergenic | - | - | None |
| [**rs8007030**](http://genome.ucsc.edu/cgi-bin/hgTracks?db=hg19&position=chr14:102399353-102399354) | 14 | 102399354 | T>A | [PPP2R5C](http://dec2013.archive.ensembl.org/Homo_sapiens/geneview?gene=ENSG00000078304) | protein coding | - | Protein Phosphatase 2 Regulatory Subunit B'Gamma | Insomnia^17^ (gene), autism^42^ (gene) |
| [**rs2241035**](http://genome.ucsc.edu/cgi-bin/hgTracks?db=hg19&position=chr16:90108831-90108832) | 16 | 90108832 | C>T | [GAS8, URAHP](http://dec2013.archive.ensembl.org/Homo_sapiens/geneview?gene=ENSG00000141013) | protein coding | intronic, 3’ downstream | Growth Arrest Specific 8 | Use of sun/UV protection*^1^ (gene) |
| [**rs2267213**](http://genome.ucsc.edu/cgi-bin/hgTracks?db=hg19&position=chr22:33863217-33863218)**■** | 22 | 33863218 | G>A | [LARGE](http://dec2013.archive.ensembl.org/Homo_sapiens/geneview?gene=ENSG00000133424) | protein coding | intronic | LARGE Xylosyl- And Glucuronyltransferase 1 | Alcohol dependence*^5^ (gene), neuroticism^2^ (gene), well-being^2^ (gene) |
| Chr: Chromosome. A1: Major allele, A2: Minor allele. For all SNP-genes highlighted in green, human tissue expression is depicted in Supplementary Figure2. ■ indicates the 7 SNPs with p<0.001 in all GWAS from set 1. Previously reported associations passing genome-wide significance (p<5x10^-8^) are marked with an asterisk. “Gene” or “SNP” in brackets indicate the origin of the reported association: previous association of one or more SNPs located in the respective gene (gene) or a direct association of the here reported SNP with the phenotype (SNP). | | | | | | | | |

| **Supplementary Table3. Top polytoxicomania-associated genes (MAGMA gene-based analysis)** | | | | | | |
| --- | --- | --- | --- | --- | --- | --- |
| **Gene** | **Chr** | **Start** | **Stop** | **# SNPs** | **Full name of gene** | **Previously reported GWAS associations (p<1x10^-5) (psychiatric phenotypes only)** |
| **CAD** | 2 | 27440258 | 27466660 | 2 | Carbamoyl-Phosphate Synthetase 2, Aspartate Transcarbamylase, And Dihydroorotase | Alcohol intake* /drinks per week*/drinks per day^1,6,7^ |
| **ROCK1** | 18 | 18529701 | 18691812 | 4 | Rho Associated Coiled-Coil Containing Protein Kinase 1 | None |
| **SLC30A3** | 2 | 27477440 | 27501093 | 1 | Solute Carrier Family 30 Member 3 | Positive affect^2^, alcohol intake*/drinks per week*/drinks per day* |
| **SLC5A6** | 2 | 27422455 | 27435175 | 2 | Solute Carrier Family 5 Member 6 | Alcohol intake*/weekly red wine intake/drinks per week*/drinks per day^1,6,7^ |
| **SMC2** | 9 | 106856213 | 106903700 | 15 | Structural Maintenance Of Chromosomes 2 | None |
| **PLA2G2E** | 1 | 20246800 | 20250110 | 1 | Phospholipase A2 Group IIE | None |
| **FAM208B** | 10 | 5726801 | 5807742 | 14 | Transcription Activation Suppressor Family Member 2 | None |
| **ACKR3** | 2 | 237469424 | 237491001 | 8 | Atypical Chemokine Receptor 3 | Age at onset of alcohol dependence^43^ |
| **OR51G1** | 11 | 4944604 | 4945569 | 4 | Olfactory Receptor Family 51 Subfamily G Member 1 (Gene/Pseudogene) | None |
| **KRTAP1-5** | 17 | 39182278 | 39183454 | 1 | Keratin Associated Protein 1-5 | None |
| **LOC100133128** | 4 | 9400867 | 9405291 | 1 | - | NA |
| Chr: Chromosome; # SNPs: Number of SNPs per gene included in analysis. Previously reported associations passing genome-wide significance (p<5x10^-8^) are marked with an asterisk. NA: Locus not listed in GWAS atlas or GWAS catalogue. | | | | | | |

**References**

1 Watanabe, K. *et al.* *Nat Genet* **51**, 1339-1348 (2019).

2 Baselmans, B. M. L. *et al.* *Nat Genet* **51**, 445-451 (2019).

3 Nagel, M. *et al.* *Nat Genet* **50**, 920-927 (2018).

4 Luciano, M. *et al.* *Nat Genet* **50**, 6-11 (2018).

5 Wang, J. C. *et al.* *Mol Psychiatry* **18**, 1218-1224 (2013).

6 Liu, M. *et al.* *Nat Genet* **51**, 237-244 (2019).

7 Karlsson Linner, R. *et al.* *Nat Genet* **51**, 245-257 (2019).

8 Kichaev, G. *et al.* *Am J Hum Genet* **104**, 65-75 (2019).

9 Gelernter, J. *et al.* *Biol Psychiatry* **77**, 493-503 (2015).

10 Nagel, M. *et al.* *Nat Commun* **9**, 905 (2018).

11 Pardinas, A. F. *et al.* *Nat Genet* **50**, 381-389 (2018).

12 Service, S. K. *et al.* *Transl Psychiatry* **2**, e116 (2012).

13 Middeldorp, C. M. *et al.* *J Am Acad Child Adolesc Psychiatry* **55**, 896-905 e896 (2016).

14 Pasman, J. A. *et al.* *Nat Neurosci* **21**, 1161-1170 (2018).

15 Goes, F. S. *et al.* *Am J Med Genet B Neuropsychiatr Genet* **168**, 649-659 (2015).

16 Ikeda, M. *et al.* *Schizophr Bull* **45**, 824-834 (2019).

17 Jansen, P. R. *et al.* *Nat Genet* **51**, 394-403 (2019).

18 International League Against Epilepsy Consortium on Complex Epilepsies. Electronic address, e.-a. u. e. a. *Lancet Neurol* **13**, 893-903 (2014).

19 Li, Q. *et al.* *Pharmacogenet Genomics* **27**, 7-18 (2017).

20 Smith, A. H. *et al.* *Alcohol Clin Exp Res* **42**, 2337-2348 (2018).

21 Lane, J. M. *et al.* *Nat Genet* **51**, 387-393 (2019).

22 Alemany, S. *et al.* *Am J Med Genet B Neuropsychiatr Genet* **168**, 459-470 (2015).

23 Fabbri, C. *et al.* *Prog Neuropsychopharmacol Biol Psychiatry* **65**, 17-24 (2016).

24 Okbay, A. *et al.* *Nat Genet* **48**, 624-633 (2016).

25 Duncan, L. *et al.* *Am J Psychiatry* **174**, 850-858 (2017).

26 Fabbri, C. *et al.* *Pharmacogenomics J* **18**, 413-421 (2018).

27 Jia, H. *et al.* *BMC Genet* **20**, 61 (2019).

28 Jones, S. E. *et al.* *PLoS Genet* **12**, e1006125 (2016).

29 Jones, S. E. *et al.* *Nat Commun* **10**, 343 (2019).

30 Hall, L. S. *et al.* *Transl Psychiatry* **8**, 9 (2018).

31 Dashti, H. S. *et al.* *Nat Commun* **10**, 1100 (2019).

32 Zhou, H. *et al.* *JAMA Psychiatry* **74**, 1234-1241 (2017).

33 Amin, N. *et al.* *Mol Psychiatry* **17**, 1116-1129 (2012).

34 Hart, A. B. *et al.* *PLoS One* **7**, e42646 (2012).

35 Anney, R. J. *et al.* *Am J Med Genet B Neuropsychiatr Genet* **147B**, 1369-1378 (2008).

36 Cox, J. W. *et al.* *J Clin Med* **9** (2020).

37 Schormair, B. *et al.* *Nat Genet* **40**, 946-948 (2008).

38 Speed, D. *et al.* *Hum Mol Genet* **23**, 247-258 (2014).

39 Anttila, V. *et al.* *Nat Genet* **45**, 912-917 (2013).

40 Agrawal, A. *et al.* *Addict Biol* **16**, 514-518 (2011).

41 Ji, Y. *et al.* *Br J Clin Pharmacol* **78**, 373-383 (2014).

42 Anney, R. *et al.* *Hum Mol Genet* **19**, 4072-4082 (2010).

43 Kapoor, M. *et al.* *Drug Alcohol Depend* **142**, 56-62 (2014).
