# Supplementary material for: Preadult polytoxicomania—strong environmental underpinnings and first genetic hints
Source: Mol Psychiatry. 2021 Apr 7;26(7):3211–22. doi: 10.1038/s41380-021-01069-2 (PMC8505259; doi:10.1038/s41380-021-01069-2)
Supplement: Supplementary file 2 — Supplementary Figures 1-4 [file 41380_2021_1069_MOESM2_ESM.pdf]

SUPPLEMENTARY FIGURES

Preadult polytoxicomania – strong environmental underpinnings and first genetic hints

Agnes A. Steixner-Kumar[1], Vinicius Daguano Gastaldi[1], Jan Seidel[1], Albert Rosenberger[2], Martin Begemann[1,3], and Hannelore Ehrenreich[1]*

[1]Clinical Neuroscience, Max Planck Institute of Experimental Medicine, Göttingen, Germany
[2] Department of Genetic Epidemiology, University Medical Center, Georg-August-University, Göttingen, Germany
[3]Department of Psychiatry and Psychotherapy, University Medical Center, Georg-August-University, Göttingen, Germany

Running head: Origins of polytoxicomania

Key words: Multiple drug use, schizophrenia, environmental risk, cannabis, alcohol, genome-wide association study (GWAS), phenotype-based genetic association study (PGAS), suicidality, autism

***Correspondence:**
Prof. Hannelore Ehrenreich, MD, DVM
Clinical Neuroscience
Max Planck Institute of Experimental Medicine
Hermann-Rein-Str.3
37075 Göttingen, GERMANY
Phone +49-551-3899628
Fax +49-551-3899670
E-Mail: **ehrenreich@em.mpg.de**

This Supplementary Figure file contains:

Supplementary Figures1-4

***Please note*: In addition to this file, a Supplementary Tables file is available, containing:**
Supplementary Tables1-3 showing prevalence of illicit drug use and listing top SNP and gene (MAGMA) results.

Steixner-Kumar et al Supplementary Figure1

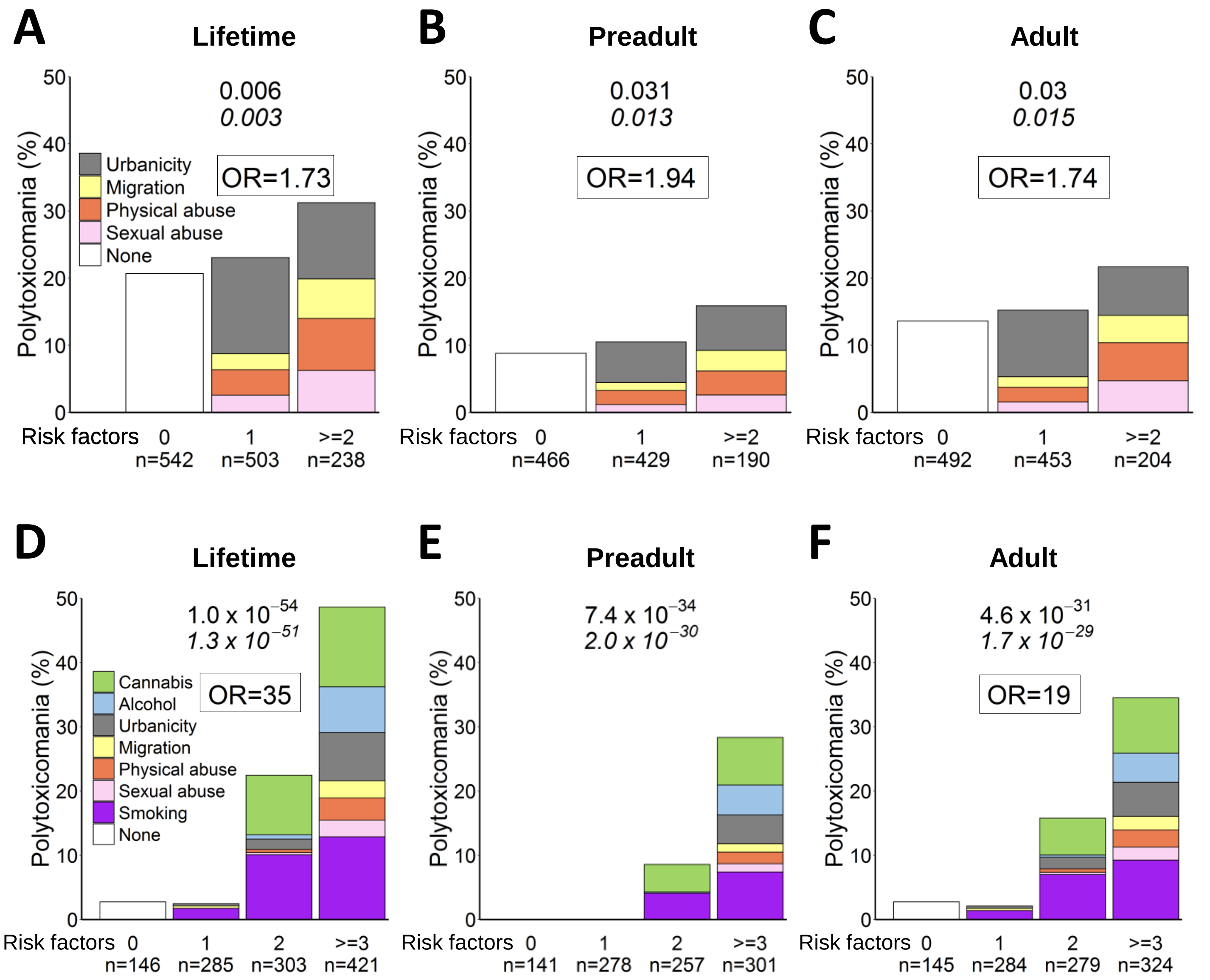

Supplementary Figure1: (A-C) Accumulation model of 4 risk factors (i.e. excluding cannabis and alcohol) as predictors of lifetime (A), preadult (B) and adult (C) polytoxicomania. **(D-F)** Accumulation model including smoking in an exploratory fashion (despite multicollinearity) as additional risk factor, i.e. 7 risk factor model, of lifetime (D), preadult (E) and postadult (F) polytoxicomania. Please note that statistics in D-F are just provided for orientation. These statistics should be interpreted with caution due to multicollinearity amongst risk factors (tight association of smoking and cannabis) and thus violation of independency assumptions. Therefore, also no OR was calculated for E (0 polytoxicomanic individuals in lowest risk group requiring permutation). Chi²-test p-values (two-sided) on top of graph, Cochran-Armitage test p-values (two-sided) underneath in italics. OR: Odds Ratio.

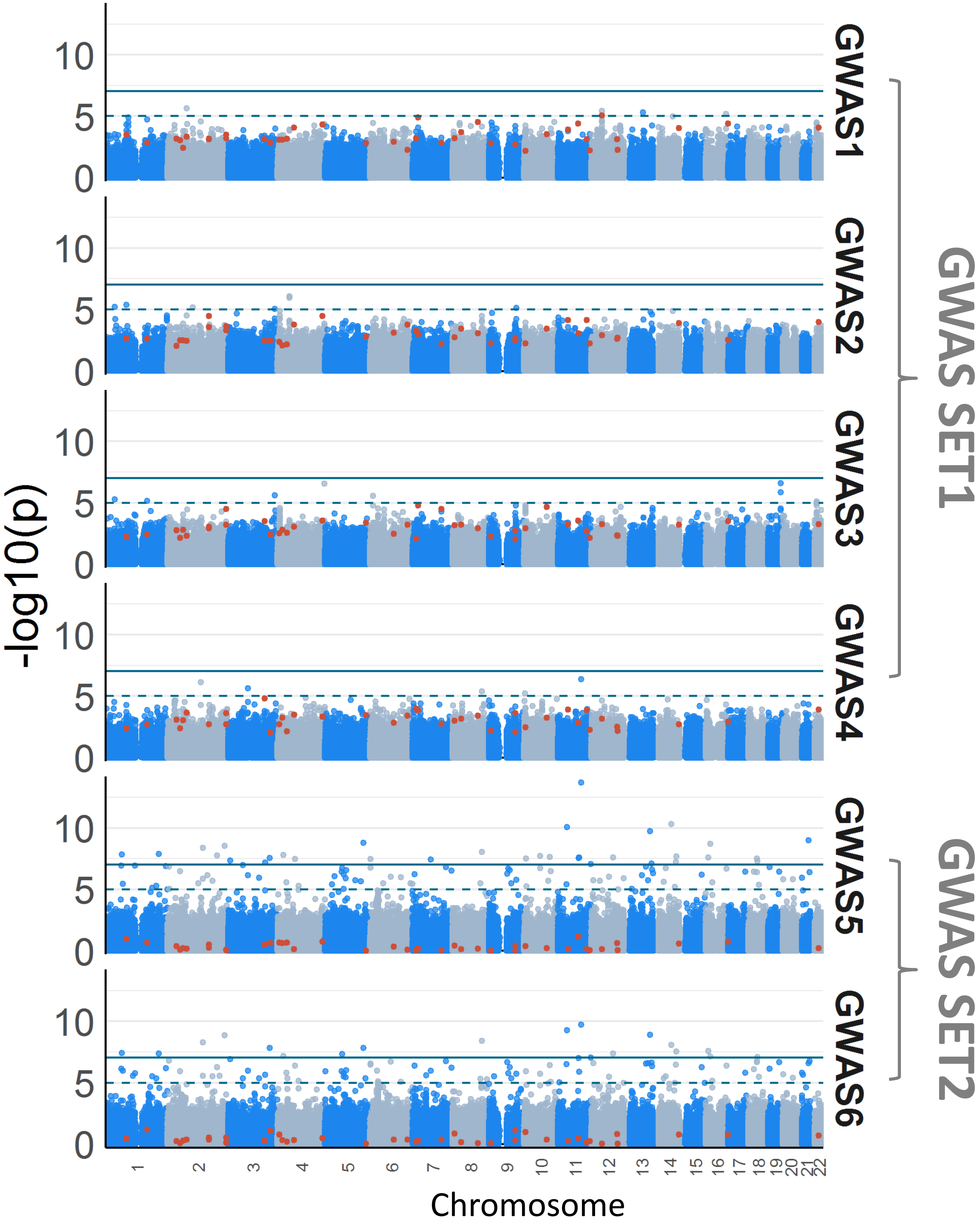

Supplementary Figure2:
Manhattan plots showing results from GWAS1-6. Final 41 top SNPs with $p<0.01$ in GWAS1-4 and $p>0.05$ in GWAS5-6 highlighted in red. Solid blue line indicates genome wide significance level ($p<9.4 \times 10^{-8} = 0.05/530,316$). Dashed blue line indicates suggestive significance level ($p<1 \times 10^{-5}$).

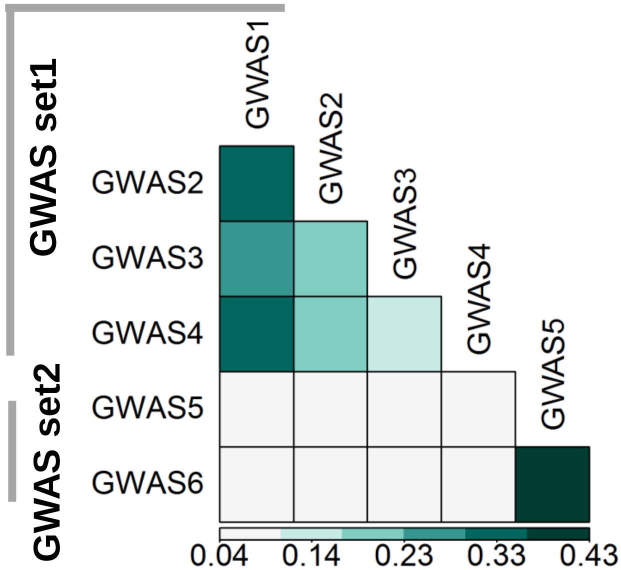

Supplementary Figure3:
Jaccard index matrix shows similarity between raw results (p<0.05) from GWAS1-6. Note that GWAS1-4 (GWAS set1) form one similarity cluster, whereas GWAS5-6 (GWAS set2) form another similarity cluster.

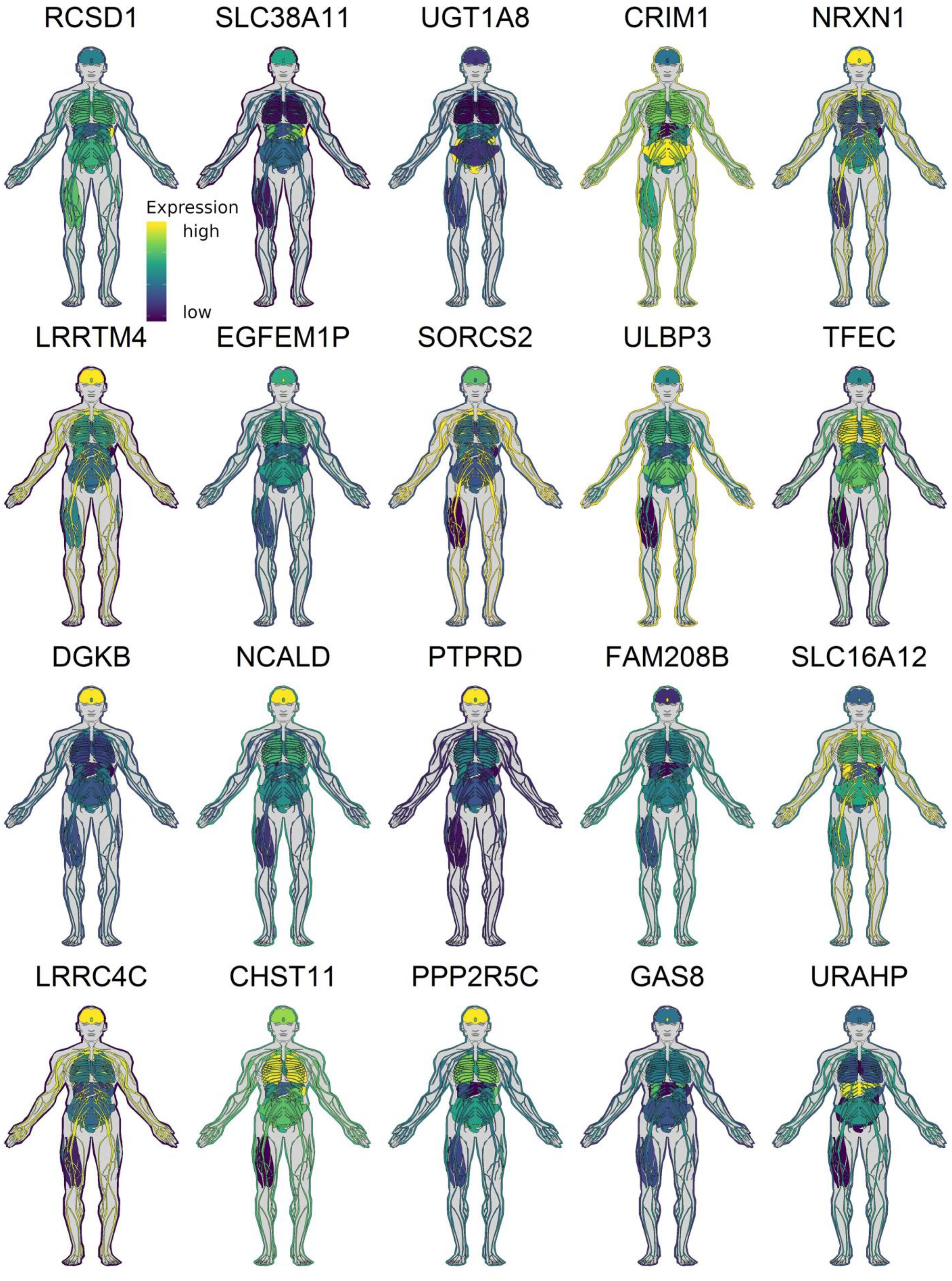

Supplementary Figure4:
Illustration of gene expression profiles. Human gene expression of the 20 genes in which top polytoxicomania-associated SNPs are located (expression data downloaded from Harmonizome). Genes correspond to the SNPs marked in green in Supplementary Table1.
